# Activation of STAT3 in Human Gastric Cancer Cells via Interleukin (IL)-6-Type Cytokine Signaling Correlates with Clinical Implications

**DOI:** 10.1371/journal.pone.0075788

**Published:** 2013-10-07

**Authors:** Zhengguang Wang, Xiulian Si, Aman Xu, Xiangning Meng, Shile Gao, Yijun Qi, Liang Zhu, Tuanjie Li, Weiping Li, Liuyi Dong

**Affiliations:** 1 Key Laboratory of Antiinflammatory and Immunopharmacology, Department of Pharmacology, Ministry of Education, Key Laboratory of Chinese Medicine Research and Development, State Administration of Traditional Chinese Medicine, Anhui Medical University, Hefei, Anhui, China; 2 Department of Surgery, the First Affiliated Hospital of Anhui Medical University, Hefei, Anhui, China; Memorial Sloan Kettering Cancer Center, United States of America

## Abstract

**Background:**

The signal transducers and activators of transcription 3 (STAT3) signaling pathway plays important roles in oncogenesis, angiogenesis, immunity, and tumor cell invasion. In the present study, we investigated the association of interleukin (IL)-6/STAT3 signaling pathway with T lymphocytes and clinical implication in patients with gastric cancer.

**Methods:**

Seventy one patients who underwent gastrectomy due to gastric adenocarcinoma were studied. Blood samples were collected before and after surgical gastrectomy to quantify the levels of IL-6, IL-10 and VEGF using an enzyme-linked immunosorbent assay, as well as T lymphocyte subsets (CD3^+^, CD4^+^, CD8^+^, CD4^+^/CD8^+^) and natural killer (NK) cells by a flow cytometry. Furthermore, the expression of IL-6, survivin, STAT3, STAT3 phosphorylation (p-STAT3), and VEGF were determined in human gastric cancer and adjacent normal mucosa through Western blot and immunohistochemistry.

**Results:**

Postoperative levels of IL-6, IL-10 and VEGF in serum were significantly lower than preoperative levels. Percentages of T-cell subsets and NK cells in blood were significantly increased after postoperative-week 1 as compared to preoperative group, which was further augmented at 1 month after gastrectomy. In addition, the expression of IL-6, survivin, STAT3, p-STAT3, and VEGF were increased in human gastric cancer tissues as compared to adjacent normal mucosa. Their expression was associated with TNM stage of gastric cancer. The level of STAT3 activation in clinical samples was correlated with IL-6 expression. All gastric tumor samples, which expressed p-STAT3, also expressed IL-6 with weak expression detected in adjacent normal mucosa.

**Conclusion:**

Increased IL-6-induced activation of STAT3 was observed in neoplastic gastric tissue, which positively correlated with tumor progression. Moreover, IL-6 and STAT3 downstream signals such as IL-10 and VEGF were reduced in patients after removal of gastric cancer as compared to pre-operation. Therefore, inhibition of the IL-6/STAT3 signaling pathway may provide a new therapeutic strategy against gastric cancer.

## Introduction

Gastric cancer is the second most common cause of cancer-related deaths, and nearly 1 million new cases are diagnosed worldwide each year [1]. The overall survival rate is not significantly improved, although early diagnosis and therapeutic treatments are in development. This is in part due to incomplete understanding of mechanism underlying tumorigenesis and metastasis of gastric cancer.

Signal transducer and activator of transcription 3 (STAT3) is a transcription factor, which can be activated by tyrosine phosphorylation in response to growth factors and cytokines (i.e., interleukin [IL]-6). IL-6, which originally was characterized as a B cell differentiation-inducing cytokine, is a cytokine that play important roles in a plethora of biological functions including inflammation, plasmacytoma genesis, immunoglobulin production by mediating cell growth, cell proliferation, and cell survival [2-4]. Depending upon the cell type, IL-6 has the ability to act through different classic protein kinase pathways, including mitogen activated protein kinase (MAPK) and phoshatidylinositol-triphosphate kinase (PI-3 kinase) [5]. IL-6 binds to the IL-6 receptor (IL-6R), which associates with gp130, therefore activates transcription factors STAT1 and STAT3, via the Janus-associated kinase (JAK), leading to severe unexpected consequences in neoplastic growth [6,7]. Upon activation, STAT3 rapidly translocates into nucleus, and binds to recognition sequence in the promoter of target genes (e.g., cyclin D1, Bcl-2, Bcl-xL, matrix metalloproteinases and vascular endothelial growth factor [VEGF]), thereby increasing their transcription [8-10]. All these target genes are implicated in regulation of cell survival, angiogenesis, immune evasion, and inflammation in tumor microenvionment.

STAT3 activation contributes to growth stimulation, anti-apoptosis, and angiogenesis, which is signiﬁcantly associated with inflammation, immunity, and oncogenesis [7,11,12]. Consequently, constitutive activation of STAT3 is responsible for a variety of human cancers, including ovarian cancer, breast cancer, leukemia, prostate cancer, head and neck cancer, and pancreatic cancer [13-17]. Blockade of the JAK/STAT3 signal may inhibit the growth of human cancers [17]. Recently, STAT3 has been demonstrated to play a pivotal role in the maintenance of gastric cancer cells survival [18-22]. Constitutive activation of STAT3 is predictive of poor prognosis in human gastric cancer [23-25]. However, it still remains unknown how activated STAT3 via interleukin (IL)-6-type cytokine signaling associates with T lymphocyte alteration during the progression of human gastric cancer. The purpose of this study was to determine whether IL-6/STAT3 signaling pathway associates with T lymphocyte changes, and correlates with the progression of human gastric cancer.

## Materials and Methods

### Patients, Experimental Design and Treatment

This investigation complies with the regulations stipulated by Anhui Medical University Ethical Committee, which follows the protocol outlined in WMA Declaration of Helsinki-Ethical Principles for Medical Research Involving Human Subjects. All patients were screened and treated for the purpose of the study at the Affiliated Hospital of Anhui Medical University, Hefei, China,and signed an informed consent form. The human ethics guidelines in the clinical research project No. kj2011Z210 (Grants from Educational Commission of Anhui Province, China, PI: Dr. Zhengguang Wang) was discussed and approved by the Human Ethics Committee in the First Affiliated Hospital of Anhui Medical University on March 25, 2011. All enrolled patients underwent total or subtotal gastrectomy. After surgery, each patient received four cycles or more of chemotherapy (chemotherapy regimens: FOLFOX4 program).

Eligible patients were adults (18 years old to 75 years old) with biopsy-confirmed gastric adenocarcinoma, who had previously completed at least four cycles of chemotherapy. According to a random procedure (1:1 ratio) into the research, all patients had normal hepatic, renal and bone marrow function (described as white cell count ≥3.5 × 10^9^ cells/L, platelets ≥80×10^9^ cells/L, total bilirubin ≤ 2 × Upper Limit of Normal, hemoglobin ≥9.0 g/dL and creatinine ≤170 µmol/L), and ECOG performance status between 0-2. Patients were excluded for serious disorders, peripheral neuropathy (NCI-CTC1 level and above), pregnancy, or breast-feeding. Patients that had verified distant metastases were excluded.

### Clinical Monitoring

Responses of curative therapy were evaluated by immune function at, before and after each treatment. Cancer patient’s quality of life score out of 60 points, where a poor quality of life was less than 20 points, and poor for 21 to 30 minutes, usually for 31 to 40 points, good for 51 to 60 minutes.

### Serum samples

Peripheral serum (5 ml) was collected before treatment and postoperative week 1 as well as after a month. Serum was centrifuged at 2000 rpm per min for 10 minutes, and the upper fluid (serum) was collected.

### Determination of serum cytokines

All blood samples without EDTA were centrifuged immediately, and then supernatants were all stored at −80°C until assayed. The serum concentrations of human IL-10, IL-6 and VEGF were quantified by an enzyme-linked immunosorbant assay (ELISA) according to the manufacturer’s instructions. The kits of IL-10, IL-6 and VEGF were provided by R&D Systems (R&D Systems, CA).

### Determination of T lymphocyte subsets

T lymphocyte subsets in blood were determined using a flow cytometer [26,27]. The identification of T-cell subsets were based on the expression of CD3, CD4, and CD8, Briefly, 100 µl of EDTA anti-coagulated blood was incubated with 20 µl of relevant monoclonal reagent containing anti-CD4-FITC, anti-CD8-PE, and anti-CD3-PE-Cy5 (Beckman Coulter), followed by adding 2 ml of erythrocytes lysis solution. Samples were protected from light at room temperature for 10 min. Leukocyte cell surface integrity was maintained by a gentle, no-wash erythrocyte lysing method. The mixture was centrifuged at 4 °C for 12000 rpm/min for 5 min and the supernatant was removed. The cell pellets were washed with phosphate-buffered saline (PBS) solution and collected by centrifugation. Finally, the sample was resuspended in 1 ml of PBS, and measured with a FACSCalibur™ flow cytometer. The sample acquisition and analysis were obtained by flow cytometer and a fully automated software-reagent combination.

### Immunohistochemistry

Sections (5 µm thick) of formalin-fixed, paraffin-embedded primary gastric specimens from gastrectomy were stained with an anti-STAT3 antibody (STAT3 F-2: sc-8019, Santa Cruz Biotechnology, Santa Cruz, CA, USA) [22,25]. The levels of VEGF, survivin, and IL-6 in the gastric samples were determined by an anti-VEGF antibody (1:100 dilution; Santa Cruz Biotechnology), an anti-survivin antibody (1:100 dilution; Santa Cruz Biotechnology), and an anti-IL-6 antibody (1:50 dilution; Santa Cruz Biotechnology), respectively. Negative controls were tissue sections immunostained with nonspecific IgG antibody. Specific antibody staining was visualized using a diaminobenzidine substrate kit. The slides were observed under a bright-field microscope. And then analyzed the integral optical density (OD) value of all images, relative protein expression levels were quantiﬁed densitometrically and expressed in the mean optical density (MOD) units.

### Western blot analysis

Whole tissue lysates were prepared from human gastric tissues with RIPA buffer. Western blotting was performed using an anti-IL-6, anti-VEGF, anti-survivin, anti-STAT3 and phosphorylated STAT3 (Tyr705) antibodies (Santa Cruz Biotechnology, Santa Cruz, CA) [22]. Proteins were detected using the enhanced chemiluminescence system according to the manufacturer’s instructions (Tanon 4500, Shandong Aibo technology Co., China). Equal loading of the sample was determined by quantitation of protein as well as by reprobing membranes for β-actin as a housekeeping protein.

### Statistical analysis

Data were expressed as mean±SD. All statistical analysis was performed in SPSS 15.0 software package. The comparison of multiple groups were analyzed with one-way analysis of variance (ANOVA) followed by LSD test and Dunnett test. *P*<0.05 was considered statistically significant.

## Results

### Demographic characteristics of patients

Eight one patients were recruited for the study, and 10 cases were withdrawn due to failure of follow up and death. As shown in Table 1, forty six patients were male and twenty five patients were female. By the T value, 8 cases were included in T1, 12 cases in T2, 7 cases in T3, and 54 cases in T4. Histopathological observations showed 62 cases exhibited regional lymph node invasion and 9 cases hade no lymph node metastasis. According to TNM staging, 5 patients were classified as stage I, 7 patients as stage II, 8 patients as stage III and 51 patients as stage IV.

**Table 1:** Demographic characteristics of the 71 gastric patients.

	n	Percentage
Sex		
Male	46	64.8
Female	25	35.2
Age (years)		
<50	15	21.1
≥50	56	78.9
Primary tumor site		
Gastric cardia	33	46.5
Gastric antrum	17	23.9
Gastric body	19	26.8
Gastric fundus	2	2.8
Diameter of tumor		
<5 cm	24	33.8
≥5 cm	47	66.2
Adenocarcinoma		
Moderately differentiated	23	32.4
Poorly differentiated	48	67.6
T stage		
T1	8	9.9
T2	12	14.8
T3	7	8.6
T4	54	66.7
TNM stage		
I	5	7.0
II	7	9.9
III	8	11.3
IV	51	71.8
Lymph node metastasis		
Present	62	87.3
Absent	9	12.7
Length of postoperative treatment		
<3w	53	74.6
≥3w	18	25.4
Extent of gastrectomy		
Total gastrectomy	49	69.0
Subtotal gastrectomy	22	31.0

### Analysis of Serum Samples Quantifying IL-6, IL-10 and VEGF

In this study, 71 patients were eligible. Before and at the end of each surgery, the patients’ serum samples were quantified for cytokine levels. In this study, the levels of IL-6, IL-10 and VEGF were quantified to determine any significant difference between cytokine concentrations in serum preoperatively and postoperatively. Table 2 showed significant reduction of cytokine concentration of IL-6, IL-10 and VEGF in postoperative serum samples as compared to preoperative levels (p<0.05). The levels of these cytokines were further decreased in postoperative month 1 when compared with those in postoperative week 1 (Table 2). These results suggest that the levels of IL-6, IL-10 and VEGF are reduced after gastrectomy.

**Table 2:** Comparison of serum cytokine levels of IL-6,IL-10 and VEGF in gastric cancer patients.

Variable	n	IL-6 (ng/L)	IL-10 (ng/mL)	VEGF (ng/L)
Preoperative	23	498.9±90.7	983.6±212.0	8347.8±1937.4
Postoperative-week1	23	380.2±87.5^*^	779.7±166.7^*^	7011.3±2044.8
Postoperative-month1	25	312.9±80.2^**^	731.9±144.6^*^	6275.5±2029.6^*^

Data are mean ± SD. Compared with the preoperative group,^* *^P<0.01,^*^P<0.05.

### Analysis of T-Cell Subsets in Patients and Controls

The percentages of T-cell subsets were analyzed in blood of a cohort of gastric cancer patients before and after gastrectomy using flow cytometry. We found that percentages of T-cell subsets (CD3^+^, CD4^+^), and CD4^+^/CD8^+^ ratio as well as NK cells were significantly increased after postoperative-week 1, which was further augmented in postoperative-month 1 as compared to those before gastrectomy (Table 3). However, the percentage of CD8^+^ was decreased at 1 month after removal of gastric cancer (Table 3). These results indicate that the immune state is improved after removal of gastric cancer.

**Table 3:** Comparison of T lymphocyte subsets during different time in gastric cancer patients.

Variable	n	CD3^+^ (%)	CD4^+^ (%)	CD8^+^ (%)	CD4^+^/CD8^+^	NK
Preoperative	23	50.5±6.4	31.6±7.2	27.8±7.3	1.1±0.6	26.9±8.1
Postoperative-week1	23	54.3±7.6	36.8±8.1^*^	24.3±8.4	1.5±0.9^*^	30.4±9.3
Postoperative-month1	25	62.6±5.4^**^	42.1±6.3^**^	22.5±7.9^*^	1.9±0.8^**^	34.2±7.9^**^

Data are mean ± SD. Compared with the preoperative group, ^**^P<0.01, ^*^P<0.05.

### Expression and cellular distribution of IL-6, survivin STAT3, and VEGF in gastric cancer tissues

**Figure 1:**
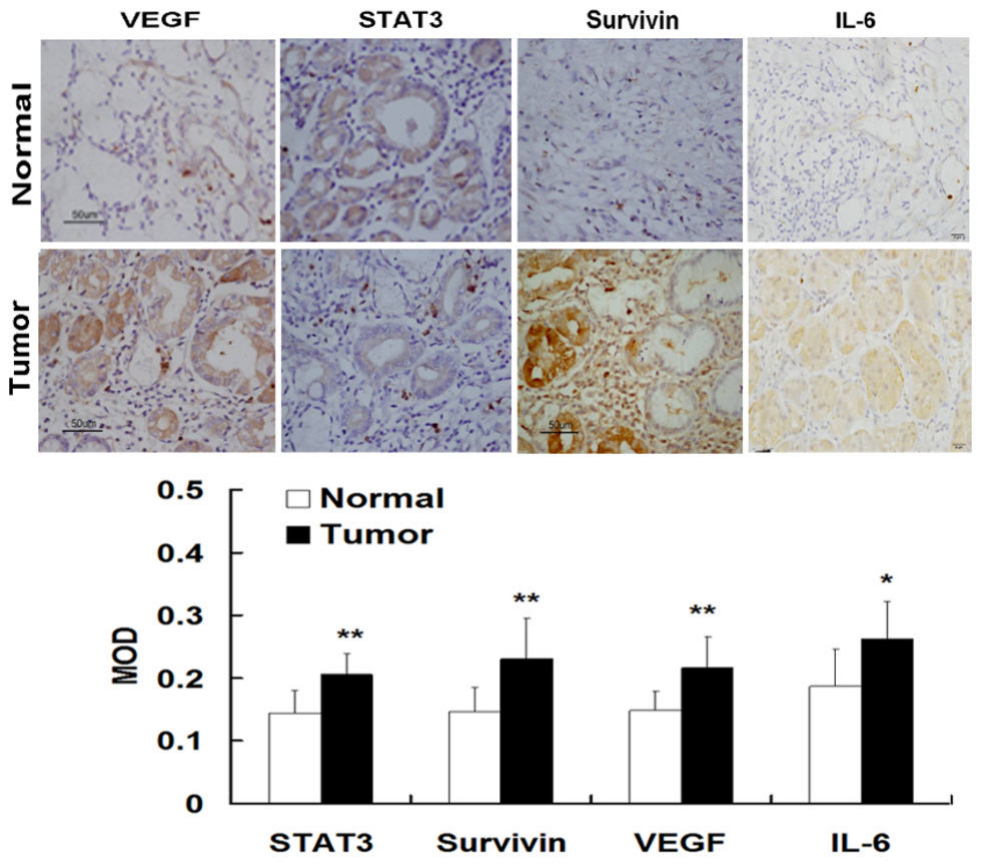
Expression of STAT3 in human gastric cancer tissues. The expression and localization of STAT3, IL-6, VEGF, and survivin in gastric cancer cells were determined using immunohistochemical staining. There was weak or negative expression of STAT3 in adjacent normal mucosa. However, there was strong expression of phosphorylated STAT3 in gastric cancer tissues. The STAT3 staining was mainly localized in the nuclei of tumor epithelial cells, which was indicated by numerous yellowish granules. STAT3 overexpression was associated with with increased expression of IL-6, surviving, and VEGF as well as with increased vessel density (Original magnification of A1-A3 and B1-B3, ×400; A4 and B4, ×200)..

The expression and cellular distribution of IL-6, survivin, STAT3 and VEGF in gastric tissues and adjacent normal mucosa of patients with gastrectomy were examined using immunohistochemical staining. It was found that the expreesion of STAT3 was increased in the foci of gastric cancer tissues as compared to its weak expression of STAT3 in adjacent normal mucosa (Figure 1). The STAT3 staining was mainly localized in the nuclei of gastric cancer cells. Similarly, the expression of IL-6, surviving and VEGF was also highly increased in gastric cancer tissues as compared to those in adjacent normal mucosa (Figure 1).

**Figure 2:**
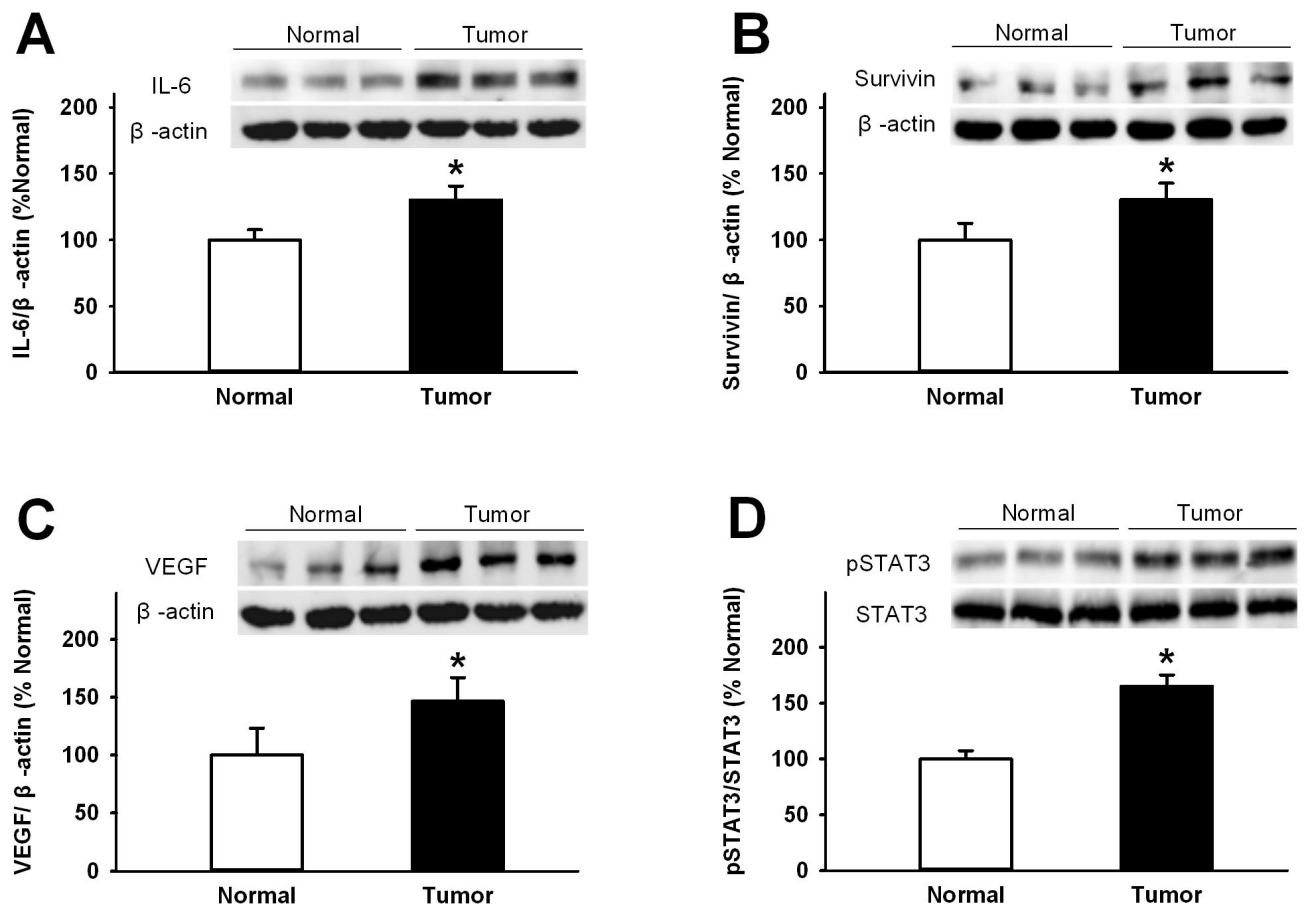
Western blot analysis of protein expression of IL-6, p-STAT3, survivin, STAT3, and VEGF. Protein levels of IL-6, Survivin, p-STAT3, STAT3, and VEGF in normal gastric and tumor tissue were determined using western blotting. Beta-actin was a loading control. Relative protein expression of IL-6 (A), VEGF (B), surviving (C), p-STAT3 (D) was normalized to of the corresponding beta-actin level. Positive immunoreactive bands were quantiﬁed densitometrically and expressed as IL-6, Survivin, p-STAT3, STAT3, and VEGF in optical density units, respectively. * P<0.01 tumor tissues versus normal gastric tissues by one-way ANOVA with post-hoc Tukey’s tests.

To further conﬁrm the increased expression of STAT3, p-STAT3, IL-6, survivin and VEGF in gastric cancer tissues, we also performed western blotting to determine their levels using anti-STAT3, anti-phosphorylated STAT3 (Tyr705), anti-IL-6, anti-survivin, anti-VEGF antibodies, respectively. As expected, a single band was observed using each antibody (Figure 2A), and the protein levels of IL-6, survivin, STAT3, p-STAT3 and VEGF in human gastric cancer tissues were all significantly higher than those in adjacent normal mucosa tissues (all P<0.001, Figure 2B). These results implicate that IL-6/ STAT3 signals are activated in gastric cancer tissues.

### IL-6/STAT3 activation correlated with TNM stage in gastric cancer

As shown in Table 4, the increased expression of STAT3 by immunohistochemistry was significantly correlated with the TNM stage of gastric cancer. Similarly, the percentage of IL-6, surviving, and VEGF expression was significantly increased in higher TNM stage as compared to low TNM stage of gastric cancer (Table 4). These results demonstrate that activation of IL-6/STAT3 pathway is associated with TNM stage of gastric cancer.

**Table 4:** Correlation between STAT3, survivin, IL-6, VEGF expression and TNM stages in gastric cancer patients.

	STAT3		*P* value	Survivin		*P* value	IL-6		*P* value	VEGF		*P* value
	**+**	**-**		**+**	**-**		**+**	**-**		**+**	**-**	
Normal gastric tissue	4 (13.3%)	26		9 (30%)	21		3 (10%)	27		2 (6.7%)	28	
Gastric cancer tissue I	2 (40%)	3		4 (80%)	1		1 (20%)	4		3 (60%)	2	
II	5 (71.4%)	2	P<0.05	7 (100%)	0	P<0.05	4 (57.1%)	3	P<0.05	5 (71.4%)	2	P<0.01
III	7 (87.5)	1		6 (75%)	2		7 (87.5%)	1		7 (87.5%)	1	
IV	46 (90.2%)	5		51 (100%)	0		47 (92.2%)	4		44 (86.2%)	7	

## Discussion

IL-6 and its downstream signals, such as STAT3, play essential roles in the process of inﬂammation and aberrant immunity as well as carcinogenesis [28-30]. STAT3 is activated for a few seconds or hours and then is deactivated to maintain homeostasis under normal circumstance. However, STAT3 activation continues, which triggers oncogene transcription under abnormal conditions [25,31]. Accumulating evidence indicates increased expression and activation of STAT3 in human gastric carcinoma [18-25,32]. It remains unclear whether increased IL-6/STAT3 activation correlates with aberrant immunity in the progression and invasion of gastric cancer. In the present study, we observed the levels of IL-6, IL-10 and VEGF in serum were significant decreased after removal of gastric cancer. This is associated with increased percentages of CD3^+^ and CD4^+^ T lymphocytes as well as NK cells. In addition, IL-6/STAT3 signals including surviving and VEGF were significantly augmented in gastric carcinoma tissues as compared to adjacent normal mucosa. Overall, our findings provide the first evidence that increased IL-6/STAT3 activation correlates with aberrant immunity, leading to the progression and invasion of gastric cancer.

IL-6 has been shown to enhance invasion of gastric cancer cells through sustained activation of STAT3 [33,34]. This is consistent with our findings that IL-6 expression was markedly associated with STAT3, and they were both over-expressed in human gastric cancer. Weak expression of both IL-6 and STAT3 was found in adjacent normal mucosa. Increased VEGF and survivin expression due to highly activation of IL-6/STAT3, helps gastric cancer cells to grow faster and to promote distant metastasis [22,35-37]. Removal of gastric cancer leads to the reduction of IL-6 and VEGF. This suggests that tumor microenvironment also plays an important role in activating IL-6/STAT3 pathway, which forms a vicious cycle to promote tumorigenesis and invasion.

Cancer immunesurveillance is an important process to eliminate tumor cells [38]. This is confirmed that increased number of T lympocytes in tumor tissues is significantly correlated with lower frequency of metastasis, recurrence and long survival, despite controversial reports exist [27,39] [34]. However, tumors have an ability to escape immunesurveillance. IL-10 is one of the immunosuppressive cytokines, which is elevated in blood in advanced gastric cancer [40-42]. This leads to inability to eliminate tumor cells in tumor microenvironment. Indeed, gastric cancer cells themselves can secrete IL-10, which may explain its reduction in patients after removal of gastric cancer as compared to pre-operation in the present study. IL-10 has a dual effect on T lymphocytes and NK cells [42-44]. This is consistent with our findings that the number of T lymphocytes and NK cells are increased in circulation system of patients after removal of gastric cancer when compared to pre-operation. Our studies also support the notion that gastric cancer patients after postoperative-week 1 still have an imbalance in their T-cell subsets, but close to normal after postoperative-month 1. However, the expression of IL-10 and infiltration of T lymphocytes (e.g., CD3^+^, CD4^+^, CD8^+^) as well as their correlation within tumor tissues are not known, which needs further investigation.

It has been shown that STAT3 activation in tumor cells can mediate an immune response by inhibiting the activity of immune cells through inflammatory cytokines probably release from invading tumor tissue [12,45]. Our data indicates that STAT3 signaling pathways play an important role in hastening immunologic tolerance. Most of the previous studies have reported that STAT3 activation in aggressive malignancies and other carcinomas is a negative prognostic factor. Our results have also demonstrated that a more aggressive clinical behavior of gastric cancer with activated STAT3, such as more frequent large vessel invasion, and lower rates of complete resectability. The roles of STAT3 in immune responses, tumor growth, and the reduction of T cell infiltration in cancer cells indicate a novel mechanism for this cellular factor [45,46]. Activation of STAT3 was a marker of positive clinical behavior. These findings indicate that STAT3 may function as a regulator of gastric cancer due to its connection with IL-6. With these data in mind and other new studies on STAT3 signaling pathway, it is worth exploring a novel STAT3-targeted treatment for gastric cancer [47-49].

In conclusion, IL-6-induced activation of STAT3 is observed in neoplastic gastric tissue, which positively correlated with tumor progression. Moreover, IL-6 and STAT3 downstream signals such as IL-10 and VEGF were reduced in patients after removal of gastric cancer as compared to pre-operation, this was associated with recovery of T lymphocytes and NK cells in peripheral circulation in these patients. Therefore, inhibition of the IL-6/STAT3 signaling pathway may provide a new therapeutic strategy against gastric cancer.

## References

[1] Ezzati M, Henley SJ, Lopez AD, Thun MJ (2005) Role of smoking in global and regional cancer epidemiology: current patterns and data needs. Int J Cancer 116: 963-971. doi:10.1002/ijc.21100. PubMed: 15880414. 15880414

[2] Hirano T, Yasukawa K, Harada H, Taga T, Watanabe Y et al. (1986) Complementary DNA for a novel human interleukin (BSF-2) that induces B lymphocytes to produce immunoglobulin. Nature 324: 73-76. doi:10.1038/324073a0. PubMed: 3491322. 3491322

[3] Teranishi T, Hirano T, Arima N, Onoue K (1982) Human helper T cell factor(s) (ThF). II. Induction of IgG production in B lymphoblastoid cell lines and identification of T cell-replacing factor- (TRF) like factor(s). J Immunol 128: 1903-1908. PubMed: 6801125. 6801125

[4] Hirano T (1998) Interleukin 6 and its receptor: ten years later. Int Rev Immunol 16: 249-284. doi:10.3109/08830189809042997. PubMed: 9505191. 9505191

[5] Yang L, Wang L, Lin HK, Kan PY, Xie S et al. (2003) Interleukin-6 differentially regulates androgen receptor transactivation via PI3K-Akt, STAT3, and MAPK, three distinct signal pathways in prostate cancer cells. Biochem Biophys Res Commun 305: 462-469. doi:10.1016/S0006-291X(03)00792-7. PubMed: 12763015. 12763015

[6] Leu CM, Wong FH, Chang C, Huang SF, Hu CP (2003) Interleukin-6 acts as an antiapoptotic factor in human esophageal carcinoma cells through the activation of both STAT3 and mitogen-activated protein kinase pathways. Oncogene 22: 7809-7818. doi:10.1038/sj.onc.1207084. PubMed: 14586407. 14586407

[7] Aaronson DS, Horvath CM (2002) A road map for those who don’t know JAK-STAT. Science 296: 1653-1655. doi:10.1126/science.1071545. PubMed: 12040185. 12040185

[8] Niu G, Wright KL, Huang M, Song L, Haura E et al. (2002) Constitutive Stat3 activity up-regulates VEGF expression and tumor angiogenesis. Oncogene 21: 2000-2008. doi:10.1038/sj.onc.1205260. PubMed: 11960372. 11960372

[9] Buettner R, Mora LB, Jove R (2002) Activated STAT signaling in human tumors provides novel molecular targets for therapeutic intervention. Clin Cancer Res 8: 945-954. PubMed: 11948098. 11948098

[10] Gamero AM, Young HA, Wiltrout RH (2004) Inactivation of Stat3 in tumor cells: releasing a brake on immune responses against cancer? Cancer Cell 5: 111-112. doi:10.1016/S1535-6108(04)00028-5. PubMed: 14998485. 14998485

[11] Bromberg J (2002) Stat proteins and oncogenesis. J Clin Invest 109: 1139-1142. doi:10.1172/JCI200215617. PubMed: 11994401. 11994401 PMC150969

[12] Yu H, Pardoll D, Jove R (2009) STATs in cancer inflammation and immunity: a leading role for STAT3. Nat Rev Cancer 9: 798-809. doi:10.1038/nrc2734. PubMed: 19851315. 19851315 PMC4856025

[13] Dolled-Filhart M, Camp RL, Kowalski DP, Smith BL, Rimm DL (2003) Tissue microarray analysis of signal transducers and activators of transcription 3 (Stat3) and phospho-Stat3 (Tyr705) in node-negative breast cancer shows nuclear localization is associated with a better prognosis. Clin Cancer Res 9: 594-600. PubMed: 12576423. 12576423

[14] Benekli M, Xia Z, Donohue KA, Ford LA, Pixley LA et al. (2002) Constitutive activity of signal transducer and activator of transcription 3 protein in acute myeloid leukemia blasts is associated with short disease-free survival. Blood 99: 252-257. doi:10.1182/blood.V99.1.252. PubMed: 11756179. 11756179

[15] Shin J, Lee HJ, Jung DB, Jung JH, Lee HJ et al. (2011) Suppression of STAT3 and HIF-1alpha mediates anti-angiogenic activity of betulinic acid in hypoxic PC-3 prostate cancer cells. PLOS ONE 6: e21492. doi:10.1371/journal.pone.0021492. PubMed: 21731766. 21731766 PMC3123343

[16] Masuda M, Suzui M, Yasumatu R, Nakashima T, Kuratomi Y et al. (2002) Constitutive activation of signal transducers and activators of transcription 3 correlates with cyclin D1 overexpression and may provide a novel prognostic marker in head and neck squamous cell carcinoma. Cancer Res 62: 3351-3355. PubMed: 12067972. 12067972

[17] Toyonaga T, Nakano K, Nagano M, Zhao G, Yamaguchi K et al. (2003) Blockade of constitutively activated Janus kinase/signal transducer and activator of transcription-3 pathway inhibits growth of human pancreatic cancer. Cancer Lett 201: 107-116. doi:10.1016/S0304-3835(03)00482-8. PubMed: 14580692. 14580692

[18] Sekikawa A, Fukui H, Fujii S, Ichikawa K, Tomita S et al. (2008) REG Ialpha protein mediates an anti-apoptotic effect of STAT3 signaling in gastric cancer cells. Carcinogenesis 29: 76-83. PubMed: 18024479. 18024479 10.1093/carcin/bgm250

[19] Giraud AS, Menheniott TR, Judd LM (2012) Targeting STAT3 in gastric cancer. Expert Opin Ther Targets 16: 889-901. doi:10.1517/14728222.2012.709238. PubMed: 22834702. 22834702

[20] Jackson CB, Giraud AS (2009) STAT3 as a prognostic marker in human gastric cancer. J Gastroenterol Hepatol 24: 505-507. doi:10.1111/j.1440-1746.2009.05822.x. PubMed: 19368630. 19368630

[21] Hsu KW, Hsieh RH, Huang KH, Li Fen-Yau A, Chi CW, et al (2012) Activation of the Notch1/STAT3/Twist signaling axis promotes gastric cancer progression. Carcinogenesis 33: 1459-1467. doi:10.1093/carcin/bgs165. PubMed: 22581828. 22581828

[22] Kanda N, Seno H, Konda Y, Marusawa H, Kanai M et al. (2004) STAT3 is constitutively activated and supports cell survival in association with survivin expression in gastric cancer cells. Oncogene 23: 4921-4929. doi:10.1038/sj.onc.1207606. PubMed: 15077160. 15077160

[23] Yakata Y, Nakayama T, Yoshizaki A, Kusaba T, Inoue K et al. (2007) Expression of p-STAT3 in human gastric carcinoma: significant correlation in tumour invasion and prognosis. Int J Oncol 30: 437-442. PubMed: 17203226. 17203226

[24] Xiong H, Du W, Wang JL, Wang YC, Tang JT et al. (2012) Constitutive activation of STAT3 is predictive of poor prognosis in human gastric cancer. J Mol Med (Berl) 90: 1037-1046. doi:10.1007/s00109-012-0869-0. PubMed: 22328012. 22328012

[25] Kim DY, Cha ST, Ahn DH, Kang HY, Kwon CI et al. (2009) STAT3 expression in gastric cancer indicates a poor prognosis. J Gastroenterol Hepatol 24: 646-651. doi:10.1111/j.1440-1746.2008.05671.x. PubMed: 19175826. 19175826

[26] Szczepanik AM, Siedlar M, Sierzega M, Goroszeniuk D, Bukowska-Strakova K et al. (2011) T-regulatory lymphocytes in peripheral blood of gastric and colorectal cancer patients. World J Gastroenterol 17: 343-348. doi:10.3748/wjg.v17.i3.343. PubMed: 21253393. 21253393 PMC3022294

[27] Yao XX, Yin L, Sun ZC (2002) The expression of hTERT mRNA and cellular immunity in gastric cancer and precancerosis. World J Gastroenterol 8: 586-590. PubMed: 12174361. 12174361 10.3748/wjg.v8.i4.586 PMC4656303

[28] Bowman T, Garcia R, Turkson J, Jove R (2000) STATs in oncogenesis. Oncogene 19: 2474-2488. doi:10.1038/sj.onc.1203527. PubMed: 10851046. 10851046

[29] Hodge DR, Hurt EM, Farrar WL (2005) The role of IL-6 and STAT3 in inflammation and cancer. Eur J Cancer 41: 2502-2512. doi:10.1016/j.ejca.2005.08.016. PubMed: 16199153. 16199153

[30] Huang S (2007) Regulation of metastases by signal transducer and activator of transcription 3 signaling pathway: clinical implications. Clin Cancer Res 13: 1362-1366. doi:10.1158/1078-0432.CCR-06-2313. PubMed: 17332277. 17332277

[31] Yu H, Jove R (2004) The STATs of cancer--new molecular targets come of age. Nat Rev Cancer 4: 97-105. doi:10.1038/nrc1275. PubMed: 14964307. 14964307

[32] Jackson CB, Judd LM, Menheniott TR, Kronborg I, Dow C et al. (2007) Augmented gp130-mediated cytokine signalling accompanies human gastric cancer progression. J Pathol 213: 140-151. doi:10.1002/path.2218. PubMed: 17724739. 17724739

[33] Lin MT, Lin BR, Chang CC, Chu CY, Su HJ et al. (2007) IL-6 induces AGS gastric cancer cell invasion via activation of the c-Src/RhoA/ROCK signaling pathway. Int J Cancer 120: 2600-2608. doi:10.1002/ijc.22599. PubMed: 17304514. 17304514

[34] Kinoshita H, Hirata Y, Nakagawa H, Sakamoto K, Hayakawa Y et al. (2013) Interleukin-6 mediates epithelial-stromal interactions and promotes gastric tumorigenesis. PLOS ONE 8: e60914. doi:10.1371/journal.pone.0060914. PubMed: 23593346. 23593346 PMC3625204

[35] Choi JH, Ahn MJ, Park CK, Han HX, Kwon SJ et al. (2006) Phospho-Stat3 expression and correlation with VEGF, p53, and Bcl-2 in gastric carcinoma using tissue microarray. APMIS 114: 619-625. doi:10.1111/j.1600-0463.2006.apm_401.x. PubMed: 16948814. 16948814

[36] Gritsko T, Williams A, Turkson J, Kaneko S, Bowman T et al. (2006) Persistent activation of stat3 signaling induces survivin gene expression and confers resistance to apoptosis in human breast cancer cells. Clin Cancer Res 12: 11-19. doi:10.1158/1078-0432.CCR-04-1752. PubMed: 16397018. 16397018

[37] Wei D, Le X, Zheng L, Wang L, Frey JA et al. (2003) Stat3 activation regulates the expression of vascular endothelial growth factor and human pancreatic cancer angiogenesis and metastasis. Oncogene 22: 319-329. doi:10.1038/sj.onc.1206122. PubMed: 12545153. 12545153

[38] Amedei A, Della Bella C, Silvestri E, Prisco D, D’Elios MM (2012) T cells in gastric cancer: friends or foes. Clin Dev Immunol: 2012: 690571 10.1155/2012/690571 PMC3369415 22693525

[39] Hong WS, Min YI, Son YS, Hong SI (1995) Peripheral blood lymphocyte subsets in patients with stomach cancer. J Korean Med Sci 10: 164-168. PubMed: 8527041. 8527041 10.3346/jkms.1995.10.3.164 PMC3054115

[40] De Vita F, Orditura M, Galizia G, Romano C, Infusino S et al. (1999) Serum interleukin-10 levels in patients with advanced gastrointestinal malignancies. Cancer 86: 1936-1943. doi:10.1002/(SICI)1097-0142(19991115)86:10. PubMed: 10570416. 10570416

[41] Fortis C, Foppoli M, Gianotti L, Galli L, Citterio G et al. (1996) Increased interleukin-10 serum levels in patients with solid tumours. Cancer Lett 104: 1-5. doi:10.1016/0304-3835(96)04213-9. PubMed: 8640735. 8640735

[42] Szkaradkiewicz A, Karpiński TM, Drews M, Borejsza-Wysocki M, Majewski P et al. (2010) Natural killer cell cytotoxicity and immunosuppressive cytokines (IL-10, TGF-beta1) in patients with gastric cancer. J Biomed Biotechnol, 2010: 2010: 901564. PubMed: 20445748 10.1155/2010/901564 PMC2860365 20445748

[43] Cai G, Kastelein RA, Hunter CA (1999) IL-10 enhances NK cell proliferation, cytotoxicity and production of IFN-gamma when combined with IL-18. Eur J Immunol 29: 2658-2665. doi:10.1002/(SICI)1521-4141(199909)29:09. PubMed: 10508240. 10508240

[44] Mocellin S, Panelli M, Wang E, Rossi CR, Pilati P et al. (2004) IL-10 stimulatory effects on human NK cells explored by gene profile analysis. Genes Immun 5: 621-630. doi:10.1038/sj.gene.6364135. PubMed: 15573087. 15573087

[45] Wang T, Niu G, Kortylewski M, Burdelya L, Shain K et al. (2004) Regulation of the innate and adaptive immune responses by Stat-3 signaling in tumor cells. Nat Med 10: 48-54. doi:10.1038/nm976. PubMed: 14702634. 14702634

[46] Burdelya L, Kujawski M, Niu G, Zhong B, Wang T et al. (2005) Stat3 activity in melanoma cells affects migration of immune effector cells and nitric oxide-mediated antitumor effects. J Immunol 174: 3925-3931. PubMed: 15778348. 15778348 10.4049/jimmunol.174.7.3925 PMC2632804

[47] Shahani VM, Yue P, Haftchenary S, Zhao W, Lukkarila JL et al. (2011) Identification of Purine-Scaffold Small-Molecule Inhibitors of Stat3 Activation by QSAR Studies. ACS Med. Chem Lett 2: 79-84. 10.1021/ml100224d PMC3021410 21243039

[48] Huang S, Chen M, Shen Y, Shen W, Guo H et al. (2012) Inhibition of activated Stat3 reverses drug resistance to chemotherapeutic agents in gastric cancer cells. Cancer Lett 315: 198-205. doi:10.1016/j.canlet.2011.10.011. PubMed: 22104727. 22104727

[49] Chen J, Wang J, Lin L, He L, Wu Y et al. (2012) Inhibition of STAT3 signaling pathway by nitidine chloride suppressed the angiogenesis and growth of human gastric cancer. Mol Cancer Ther 11: 277-287. doi:10.1158/1535-7163.MCT-11-0648. PubMed: 22203730. 22203730

